# HEXIM1, a New Player in the p53 Pathway

**DOI:** 10.3390/cancers5030838

**Published:** 2013-07-04

**Authors:** Qiao Jing Lew, Kai Ling Chu, Yi Ling Chia, Nge Cheong, Sheng-Hao Chao

**Affiliations:** 1 Expression Engineering Group, Bioprocessing Technology Institute, A*STAR (Agency for Science, Technology and Research), 20 Biopolis Way, #06-01, Singapore 138668, Singapore; 2 Department of Microbiology, National University of Singapore, Singapore 117597, Singapore

**Keywords:** HEXIM1, P-TEFb, p53, HDM2, NPM, NPMc+

## Abstract

Hexamethylene bisacetamide-inducible protein 1 (HEXIM1) is best known as the inhibitor of positive transcription elongation factor b (P-TEFb), which controls transcription elongation of RNA polymerase II and Tat transactivation of human immunodeficiency virus. Besides P-TEFb, several proteins have been identified as HEXIM1 binding proteins. It is noteworthy that more than half of the HEXIM1 binding partners are involved in cancers. P53 and two key regulators of the p53 pathway, nucleophosmin (NPM) and human double minute-2 protein (HDM2), are among the factors identified. This review will focus on the functional importance of the interactions between HEXIM1 and p53/NPM/HDM2. NPM and the cytoplasmic mutant of NPM, NPMc+, were found to regulate P-TEFb activity and RNA polymerase II transcription through the interaction with HEXIM1. Importantly, more than one-third of acute myeloid leukemia (AML) patients carry NPMc+, suggesting the involvement of HEXIM1 in tumorigenesis of AML. HDM2 was found to ubiquitinate HEXIM1. The HDM2-mediated ubiquitination of HEXIM1 did not lead to protein degradation of HEXIM1 but enhanced its inhibitory activity on P-TEFb. Recently, HEXIM1 was identified as a novel positive regulator of p53. HEXIM1 prevented p53 ubiquitination by competing with HDM2 in binding to p53. Taken together, the new evidence suggests a role of HEXIM1 in regulating the p53 pathway and tumorigenesis.

## 1. Introduction

Hexamethylene bisacetamide-inducible protein 1 (HEXIM1) was initially identified in 1999 by Kusuhara, *et al.* from vascular smooth muscle cells treated with hexamethylene bisacetamide (HMBA), an inhibitor of proliferation [1]. In the same year, Ghatpande, *et al.* cloned the HEXIM1 cDNA from the presumptive heart-forming regions of chicken embryos and named it cardiac lineage protein-1 (CLP-1) [2]. The HEXIM1/CLP-1 knockout mice were embryonic-lethal and exhibited phenotypes of cardiac hypertrophy [3,4]. HEXIM1 was also identified as a binding protein of estrogen receptor α (ERα) from a yeast two-hybrid screen using a MCF7 breast cancer cell cDNA library [5]. Estrogen was found to down-regulate HEXIM1 expression at both protein and mRNA levels. Because of this observation, HEXIM1 was also named as estrogen down-regulated gene 1 (EDG1) [5]. In 2003, research groups led by Olivier Bensaude and Qiang Zhou revealed a major biological function of HEXIM1. They demonstrated that HEXIM1 associated with positive transcription elongation factor b (P-TEFb) and inhibited its activity [6,7].

P-TEFb was identified and purified by David Price’s group based on its sensitivity to 5,6-dichloro-1-beta-D-ribofuranosylbenzimidazole (DRB), which inhibited RNA polymerase II (RNAP II) transcription at the elongation stage [8,9]. P-TEFb is a protein complex composed of cyclin-dependent kinase 9 (CDK9) and a cyclin partner (*i.e.*, cyclin T1, T2a, T2b, or K) with cyclin T1 being the predominant CDK9-associated cyclin [9,10,11]. P-TEFb phosphorylates the *C*-terminal domain of the largest subunit of RNAP II and allows the polymerase to enter the elongation phase [9,12,13]. Treatment of cells with flavopiridol, most potent and selective P-TEFb inhibiting compound, blocked 60-70% of RNAP II transcription as detected by nuclear run-on assays [14,15]. This pivotal finding clearly demonstrates that most of cellular genes are regulated by P-TEFb at the elongation stage. Furthermore, three genome-wide studies using ChIP-on-chip assays found that RNAP II occupied the promoters of most protein-coding genes in *Drosophila* and human embryonic stem cells without entering into productive elongation [16,17,18]. Such genomic distribution of poised RNAP II molecules re-confirms the significance of P-TEFb in gene expression. Transcription of many viruses is also under the control of P-TEFb. The best-studied regulation of viral transcription is Tat transactivation of human immunodeficiency virus (HIV). The HIV transactivator, Tat, recruits P-TEFb to the viral promoter through the interaction with cyclin T1, resulting in the generation of full-length viral transcripts [19,20]. A compound screening was carried out in search for the inhibitors of HIV Tat transactivation. Surprisingly, all the compounds identified were P-TEFb inhibitors, indicating an essential role of P-TEFb in controlling HIV transcription [21].

**Figure 1:**
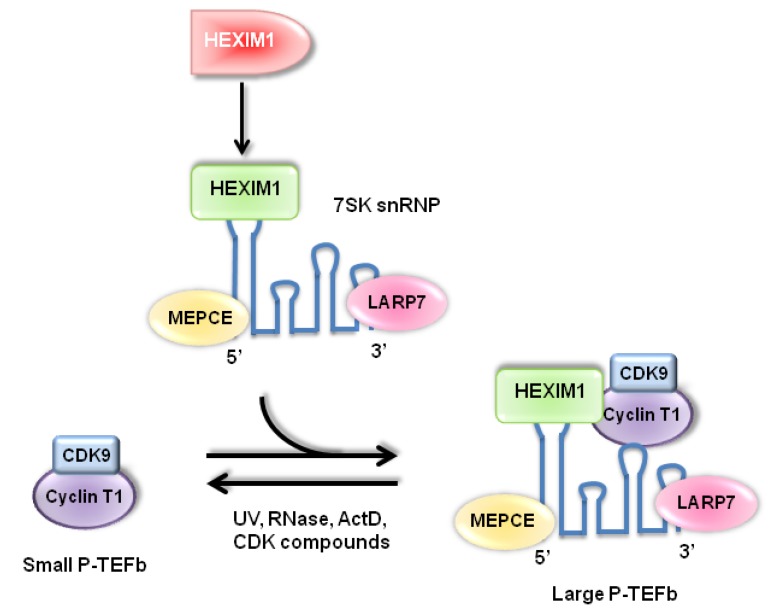
.Two P-TEFb complexes are found in cells. The small P-TEFb complex, composed of cyclin T1 and CDK9, is the active form of P-TEFb. The kinase activity of P-TEFb is inhibited when P-TEFb interacts with HEXIM1 and 7SK snRNA to form the large P-TEFb complex. Two other components of the large complex, MEPCE and LARP7, have been recently identified.

Having an estimated molecular mass of 150 kD, the P-TEFb complex consisting of CDK9/cyclin T1 was shown to exhibit kinase activity [9]. It was later reported by several groups that the CDK9-containing protein complex with a larger molecular mass was isolated through glycerol gradient sedimentation, shedding lights that two different forms of P-TEFb existed in cells [22,23]. Initially, it was unknown what caused the enzymatic inhibition of P-TEFb within the large complex [24,25]. Soon after, both 7SK small nuclear RNA (snRNA) and HEXIM1 were identified and established as the new subunits of the large P-TEFb complex [6,7,24,25]. The 7SK snRNA-bound HEXIM1 exerted an inhibitory function on P-TEFb, while neither 7SK nor HEXIM1 alone instigate any effects [7,26]. It has been proposed that association with 7SK snRNA induces the conformational change of HEXIM1 protein and makes the cyclin T binding domain of HEXIM1 more accessible for P-TEFb binding [26]. In addition, a methylphosphate capping enzyme MEPCE and a La related protein LARP7 were identified as 7SK snRNA binding proteins [27,28,29]. A model for the regulatory mechanism of the P-TEFb protein complexes by HEXIM1 is summarized in Figure 1.

HEXIM1 contains several functional domains. The *N*-terminus of HEXIM1, amino acids (a.a.) 1–150, has been characterized as a self-inhibitory domain (ID). Deletion of the ID enhances the inhibitory effects of HEXIM1 on P-TEFb activity [7,30]. The region between a.a. 150–180 of HEXIM1, which includes a stretch of basic residues, is referred to as the basic region (BR). The BR contains the binding motif for 7SK snRNA, KHRR (a.a. 152–155). When the KHRR sequence is replaced by ILAA, the mutant HEXIM1 protein fails to interact with 7SK snRNA and the formation of the large P-TEFb complex is disrupted [26]. The P-TEFb binding motif, PYNT (a.a. 202–205), is located between the BR and acidic region (AR, a.a. 210–250). In the absence of 7SK snRNA, the AR can interact with the adjacent BR. Since the P-TEFb binding motif is located between the BR and AR, the BR-AR interaction may establish an auto-inhibitory conformation which prevents the association between HEXIM1 and P-TEFb [31]. When 7SK snRNA binds to the BR, the BR-AR interaction is disrupted and the PYNT motif becomes accessible for P-TEFb binding [31]. HEXIM1 can form a homodimer or a heterodimer with a HEXIM1-related protein, HEXIM2, through the dimerization domain (DD) at the *C*-terminus of HEXIM1 [30,32,33].

Besides P-TEFb, several HEXIM1 binding proteins have been identified. HEXIM1 binds to histone deacetylases (HDACs) along with MyoD, indicating a role in regulating skeletal muscle cell differentiation [34]. Interaction between Importin α, HEXIM1, and cyclin T1 has been reported. This finding suggests a possible mechanism for HEXIM1/Importin α-mediated nucleo-cytoplasmic transport of cyclin T1 since no nuclear localization signals are present in cyclin T1 [35]. ERα is present in more than half of breast tumors and therefore, this receptor has been the most widely targeted protein in breast cancer therapy [36,37]. HEXIM1 competes with cyclin T1 in binding to ERα. When associated with HEXIM1, the transcriptional activity of ERα is inhibited, suggesting a role of HEXIM1 in breast cancer [38]. It has been shown that HEXIM1 directly interacts with the p65 subunit of NF-κB and inhibits the transcriptional activity of NF-κB [39]. In an earlier report, NF-κB was shown to recruit P-TEFb through the interaction with the p65 subunit, resulting in activation of NF-κB-dependent transcription [40]. These three-way interactions create an inevitable competition between HEXIM1 and P-TEFb in regulating the activity of NF-κB. Shimizu *et al.*, demonstrated that HEXIM1 associated with the glucocorticoid receptor (GR) in the absence of 7SK snRNA and P-TEFb and regulated the GR-mediated gene expression [41]. The significance of this study is to reveal the involvement of HEXIM1 in the P-TEFb-independent bioprocesses.

Our recent studies demonstrated the functional correlation between HEXIM1 and the p53 signaling pathway. We identified p53 as well as two important regulators of p53, nucleophosmin (NPM) and human double minute-2 protein (HDM2), as the novel HEXIM1 binding proteins. In this review, we will summarize our findings and discuss the role of HEXIM1 in cancer.

## 2. p53 and Its Regulators, HDM2 and NPM

p53 is a tumor suppressor protein which regulates cell cycle and prevents cancer genesis. As such, p53 has been described as “the guardian of the genome” because of its role in conserving stability by preventing genome mutation [42]. The human p53 protein consists of 393 amino acids with five major functional domains: transactivation (TA), proline-rich (PR), DNA-binding (DBD), oligomerization (OLI), and negative regulation (NEG) domains [43].

The *N*-terminal TA domain, containing amino acids (a.a.) 1–42, recruits the basal transcriptional machinery, such as the TATA box binding protein (TBP) and TBP-associated factors, to activate the expression of p53 target genes [44,45]. The TA domain is followed by the PR region (a.a. 63–97), which is required for p53-mediated apoptosis and suppressing tumour cell growth [46,47]. The sequence-specific DBD is located within the central part of p53 (a.a. 102–292). Most mutations that deactivate p53 in cancer usually occur within the DBD and destroy the ability of p53 binding to its target DNA sequences [48]. The tetramerization of p53 takes place in the OLI domain (a.a. 323–356). Beside its importance for DNA binding, the OLI domain is also responsible for protein-protein interactions, post-translational modifications, and protein degradation of p53 [49]. Likewise, the NEG domain located at the *C*-terminus of p53 (a.a. 360–393) is involved in its own degradation. Holding major ubiquitination sites for HDM2, the ubiquitinated p53 is directed to proteosomal degradation [50,51,52,53,54,55].

Many regulators of p53 have been identified. Here we only focus on HDM2 and NPM, two HEXIM1 binding proteins. HDM2 (or MDM2, the mouse homolog), the best-known p53 regulator, is an E3 ubiquitin ligase that targets itself and p53 for protein degradation by the proteasome [56,57]. On the contrary, NPM functions as a positive regulator of p53 in the ARF-dependent and -independent manners. ARF, a tumor suppressor, binds to MDM2 and promotes rapid degradation of MDM2, resulting in p53 stabilization and accumulation [58]. In the ARF-dependent pathway, NPM associates with ARF in high-molecular-weight complexes. NPM stabilizes ARF by retarding its turnover and leads to p53 activation [59,60]. NPM is also found to interact with HDM2 directly and protect p53 from the HDM2-mediated degradation in an ARF-independent fashion [61]. Interestingly, both HDM2 and NPM are also involved in regulation of P-TEFb activity through modulating HEXIM1. In the following chapters, we will describe the involvement of NPM and HDM2 in regulating p53 in greater detail and discuss the functional interactions between NPM, HDM2, and P-TEFb/HEXIM1.

## 3. NPM and NPMc+ Regulate P-TEFb Activity through the Interaction with HEXIM1

NPM (also known as B23, numatrin, or NO38) encoded by the *NPM1* gene is an abundant multifunctional phosphoprotein that mainly resides in nucleoli. It is required for several cellular processes such as ribosome biogenesis, cell proliferation, and transformation [62,63,64]. Apart from functioning as a histone chaperone protein in the formation of nucleosome, NPM is also involved in centrosome duplication [65,66].

The connection of NPM to cancer and the p53 pathway has extensively been demonstrated. However, it is still debatable whether NPM function as a tumor suppressor or an oncogene. As mentioned earlier, NPM not only stabilizes p53 through antagonizing HDM2 [61], but also associates and stabilizes ARF within the nucleolus, resulting in induction of p53 [67,68]. Such positive regulation of p53 has been depicted in UV-exposed cells where NPM gets up-regulated and transiently translocated from nucleolus to nucleoplasm where it interacts with HDM2 [69]. Moreover, NPM also interacts directly with p53 to enhance the stability and transcriptional activation of p53 [70]. NPM is haploinsufficient for its function which denotes *NPM^+/−^* cells to have a significant degree of genomic instability, resulting in an increased susceptibility to oncogene transformation [71]. The tumor suppressing function of NPM is suggested based on these data. On the contrary, the elevated NPM level is often observed in several tumor cells such as gastric, colon, ovarian and prostate cancer, bladder, breast cancers [72,73,74,75,76,77]. Recent studies have reported that overexpression of NPM promotes cell survival, inhibits apoptosis, and induces the migration and invasion of cancer cells [78,79,80], supporting a role of NPM as an oncogene. Taken together, all these contradicting discoveries clearly demonstrate that NPM plays an important role in tumorigenesis, either as a tumor suppressor, an oncogene, or both.

*NPM1* is one of the most frequently mutated genes in acute myeloid leukemia (AML). About 35% of AML patients carrying NPMc+, the cytoplasmic-mislocated mutant form of NPM [81]. The NPMc+ mutation is caused by an insertion of four nucleotides at the exon 12 of *NPM1* gene [82]. As a result, nucleolar localization signal (NLS) which located at the *C*-terminal of wild type NPM protein is disrupted and an additional nuclear export signal (NES) is inserted at the *C*-terminal of mutant NPM protein [83,84]. Therefore, the mutant NPMc+ protein is localized in the cytoplasm instead of nucleoli. A distinct expression profile was observed in AML bearing the NPMc+ mutation, raising the possible connection between NPMc+ and transcriptional regulation [85].

**Figure 2:**
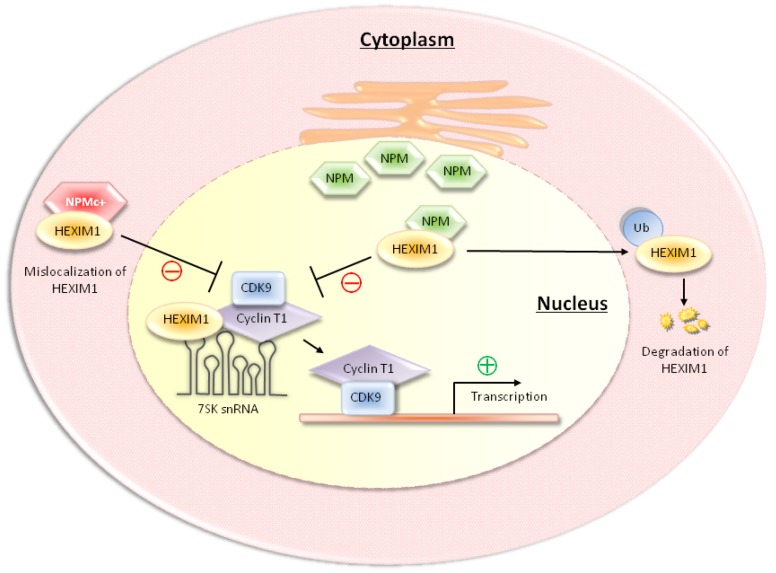
NPM binds to HEXIM1 and mediates the proteasome-dependent degradation of HEXIM1, which favors the release of the small P-TEFb (*i.e*., CDK9/cyclin T1) from the HEXIM1-containing large P-TEFb complexes. The cytoplasmic NPM mutant, NPMc+, associates and misallocates a portion of HEXIM1 in cytoplasm, resulting in decreases in the formation of large P-TEFb complexes and activation of RNAP II transcription.

In our laboratory, we identified NPM and NPMc+ as novel HEXIM1-binding proteins [86]. The functional interactions between HEXIM1 and NPM/NPMc+ are summarized in Figure 2. Overexpression of NPM induced the proteasome-mediated degradation of HEXIM1 [86]. Since HEXIM1 is required to form the large P-TEFb complex and block kinase activity of P-TEFb, a decrease in the level of HEXIM1 would influence the equilibrium between small and large P-TEFb complexes, leading to activation of the P-TEFb-dependent transcription (Figure 2). Using a green fluorescent protein (GFP) tagged NPMc+ fusion protein, we found that NPMc+ associated with HEXIM1 and sequestered a portion of HEXIM1 in the cytoplasm [86]. As a transcription factor, HEXIM1 is present in nuclei, where it regulates RNAP II transcription and P-TEFb activity. Therefore, mislocalization of HEXIM1 in the cytoplasm would decrease the formation of the large/inactive P-TEFb complexes and thereby results in higher RNAP II transcription (Figure 2).

To determine the physiological importance of our findings, we analyzed the level and sub-cellular distribution of HEXIM1 in an AML cell line carrying the NPMc+ mutation (*i.e.*, AML3 cell line). Compared to a wild-type NPM AML cell line, AML2, lower HEXIM1 protein level was detected in AML3 cells [87]. In addition, cytoplasmic localization of HEXIM1 was only observed in AML3 cells, but not in AML2 cells [86]. As expected, an increase in P-TEFb-mediated transcription was detected in AML3 cells [86]. Our results suggest the potential involvement of HEXIM1/P-TEFb in the tumorigenesis of AML bearing the NPMc+ mutation.

## 4. HDM2 Regulates P-TEFb Activity through the Ubiquitination of HEXIM1

MDM2 (or HDM2, the human homolog) is a well-studied negative regulator of p53 protein. The wild type p53 in unstressed cells appears to be an unstable protein with a very short half life due to MDM2-mediated proteasome degradation [56,57]. The *N*-terminus of MDM2 interacts with *N*-terminal transactivation domain of p53 and effectively blocks p53-mediated transactivation [88,89]. In addition, MDM2 carries the p53 specific E3 ubiquitin ligase within the *C*-terminal RING finger domain [57,90]. Upon interaction with p53, MDM2 E3 ligase, along with p300/CBP (CREB-binding protein) co-activator proteins, polyubiquitinates p53 in the nucleus [91,92,93]. This crucial step which only occurs in the nucleus would be a pre-requisite for subsequent 26S proteasome degradation [94]. MDM2 RING finger domain, but not the leucine-rich nuclear export signal (NES), is important for the relocalization of p53 out of the nucleus into the cytoplasm for proteasome degradation [95,96]. However, 26S proteasome is present in both the nucleus and the cytoplasmic compartment [97]. In parallel to cytoplasmic compartment, the nucleus proteasome also contributed to MDM2-mediated p53 degradation pathway.

Many strategies have been formulated to disrupt the MDM2-p53 interaction as the anti-cancer approaches. Chene and co-workers designed a synthetic peptide based on the X-ray structure of p53 co-crystallizing with HDM2 [98]. When used with tumor cells that overexpress HDM2, this peptide induced the death of these tumor cells by apoptosis [98]. Nutlin-3 is a small compound that mimics the interaction of p53 protein and potently competes out p53 from MDM2. Importantly, treatment with nutlin-3 also stimulates a dose-dependent increase in the expression level of p21 and anti-proliferative activities across different cell lines carrying wide type p53 [99]. Similarly, another small molecule, RITA (reactivation of p53 and induction of tumor cell apoptosis), binds directly to p53. It induces a conformational change in p53 and abolishes p53-HDM2 interaction to activate p53 [100]. Additionally, the application of antibodies against MDM2 was proposed. Microinjection of an antibody specifically targeting against the p53 binding domain of MDM2 effectively disrupts the MDM2-p53 complex formation to increase p53-dependent transcription activation [101]. The ARF protein, which is introduced formerly, plays a significant role as a tumor suppressor that blocks the MDM2-dependent p53 degradation. An ARF synthetic peptide was designed to imitate the *N*-terminal domain of ARF. Acting like ARF, this synthetic peptide was shown to bind directly onto the central acidic domain of MDM2, inhibit MDM2-dependent ubiquitination and protect p53 from ubiquitination-mediated proteasome degradation [102].

Since we have established HEXIM1 to have a connection with NPM and NPM in turn also interacts with HDM2, such close associations allow us to anticipate HEXIM1 as a new substrate for HDM2. Indeed, HDM2 ubiquitinates the lysine residues located within the BR of HEXIM1; however, the ubiquitination of HEXIM1 by HDM2 does not lead to proteasome degradation pathway [103]. To investigate the impact of ubiquitination on HEXIM1’s function, we generated the HEXIM1-ubiquitin fusion protein and examined its effect on P-TEFb-dependent transcription. Compared to the wild-type HEXIM1, the ubiquitinated HEXIM1 exhibited stronger inhibition on P-TEFb activity, suggesting a role of HDM2 in P-TEFb regulation [103]. On the other hand, the possible involvement of HEXIM1 in the p53 pathway is suggested. As a new binding protein and an enzymatic substrate of HDM2, HEXIM1 may have an impact on the p53 stability mediated through HDM2. It would be worthwhile to design a series of synthetic peptides based on the amino acid sequences of HEXIM1 BR and evaluate their effects on p53 activation.

## 5. HEXIM1 Stabilizes p53 through the Protein-Protein Interaction with p53

In the late 1980s, several discoveries have well defined p53 to be an anti-oncogenic protein. Studies have shown that cells lacking p53 progressively become tumors during excessive unregulated genomic mutations [50,52,104]. Henceforth, stabilizing p53 has been a focus as a potential remedy for cancers [50,51,52,53,104,105,106,107,108,109,53,104]. Of the many studies on positive regulators of p53, several are relevant to our discussion of HEXIM1. For instance, in 1998, An *et al.* identified hypoxia-inducible factor 1alpha (HIF-α) as p53 interacting counterpart. Under hypoxia condition, HIF-α becomes activated to stabilize and transactivate p53 [110,111]. Shortly after, Yuan and co-workers determined that the *N*-terminal region of p53 interacted with p300/CBP. Through p53-p300/CBP interaction, the p53 *C*-terminal domain gets acetylated and mediates p53 transactivation as well [112]. Herpesvirus-associated ubiquitin-specific protease (HAUSP) has been found to associate with and deubiquitinate p53, which is crucial for tumor suppression function [53,107]. The von Hippel-Lindau tumor suppressor protein (pVHL) was later found to directly interact with p53 and p300 acetylation ensued upon genotoxic stress. Moreover, acetylation thwarts the nuclear-export of p53 preventing it from HDM2 proteasome degradation and ultimately stabilizes p53 to execute cell arrest or apoptosis [108,112].

**Figure 3:**
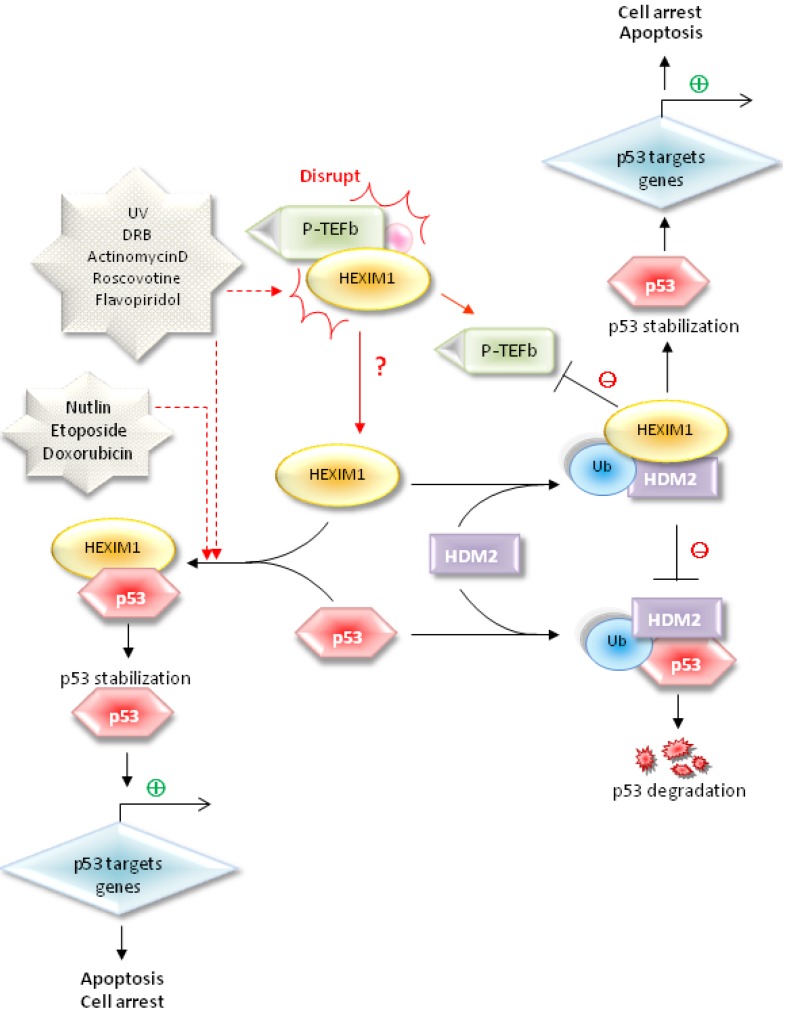
Treatments with UV radiation, flavopiridol, DRB, roscovitine, actinomycin D, and doxorubicin, etoposide, and nutlin-3 increase the HEXIM1-p53 interaction and lead to induction of p53. UV, flavopiridol, DRB, roscovitine, and actinomycin D treatments can release more “free” HEXIM1 from the large P-TEFb complexes and may further enhance the association between p53 and HEXIM1. HEXIM1 not only competes with HDM2 in binding to p53, but also interacts with HDM2, resulting in activation of p53. The HDM2-ubiquitinated HEXIM1, which is not degraded through the proteasome-mediated pathway, exerts stronger inhibition on P-TEFb activity.

**Figure 4:**
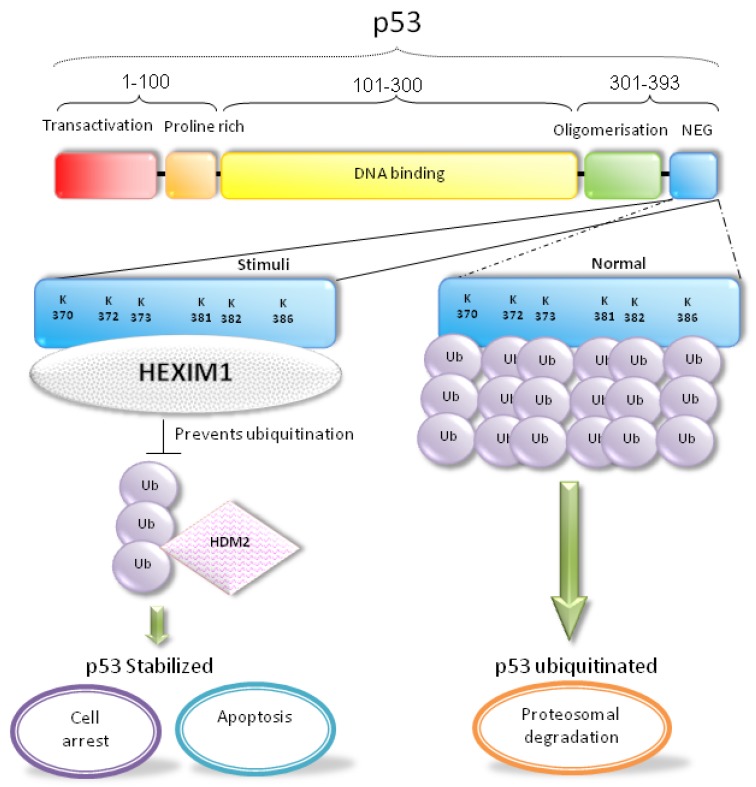
The NEG domain of p53 contains the lysine residues targeted by HDM2 for ubiquitination and degradation of p53. Domain study of p53 reveals that the NEG domain is essential for the association between HEXIM1 and p53 [113]. It is proposed that HEXIM1 binds and stabilizes p53 protein by blocking the ubiquitination of p53 by HDM2.

In our recent study, we demonstrated that HEXIM1 interacted directly with p53 protein via co-immunoprecipitation and GST pull-down assays [113]. It is through the discovery of NPM-HEXIM1 and HDM2-HEXIM1 interactions in which we chanced upon p53 to be a new partnering candidate for HEXIM1. The involvement of HEXIM1 in regulation of p53 activation is summarized in Figure 3. It is noteworthy that p53 interacts with the “free” HEXIM1 rather than the HEXIM1 present in the large inactive P-TEFb complex [113]. Domain study demonstrated that HEXIM1 BR (basic region) in cooperation with the *C*-terminus of HEXIM1 was required for sufficient p53 binding. Interestingly, we unraveled the NEG (negative regulation) domain of p53 was essential to interact with HEXIM1 [113]. With reference to Figure 4, the NEG domain consists of various lysine residues which have been well-established as the HDM2 ubiquitination sites [50,51,52,53,54,55]. Since these ubiquitination sites resided in the HEXIM1 binding domain of p53, we reckoned that HEXIM1 occupancy to the NEG domain might hinder the p53-HDM2 interaction and thereby block the ubiquitination of p53 (Figure 4). As expected, overexpression of HEXIM1 disrupts the interaction between HDM2 and p53 resulting in stabilization of p53 and activation of p53 downstream targets (such as PUMA and p21) in various cancer cell lines [113]. This finding further verify that HEXIM1 might compete with HDM2 in binding to p53 at the same site (Figure 3) [113]. Previously, we also detected the association between HDM2 and HEXIM1, resulting in ubiquitination of HEXIM1 [103]. This HEXIM1-HDM2 interaction may protect a portion of p53 from being associated with and ubiquitinated by HDM2, and indirectly contribute to the stability of p53 (Figure 3).

Remarkably, treatments with UV radiation, CDK inhibiting (flavopiridol, DRB, roscovitine), transcription inhibiting (actinomycin D), and p53 inducing compounds (doxorubicin, etoposide, nutlin-3) not only increase p53 levels [54,105,113], but also enhance the protein-protein interactions between HEXIM1 and p53 (Figure 3) [113]. UV, flavopiridol, DRB, roscovitine, and actinomycin D treatments disrupt the formation of large P-TEFb complexes, resulting in releasing more HEXIM1 from the large P-TEFb complexes. Such treatments should increase the pool of “free” HEXIM1 in cells and may contribute to the increased p53-HEXIM1 interaction (Figure 3). However, treatment with doxorubicin, etoposide, and nutlin-3, which has no effects on the formation of large P-TEFb complexes, enhances the p53-HEXIM1 association as well (Figure 3) [113]. This observation indicates that cells should have plenty of the “free” HEXIM1 to interact with p53 upon the stimulation [6,114]. Unlike doxorubicin and etoposide (topoisomerase II inhibitors), treatment with camptothecin, an topoisomerase I inhibitor and a p53 inducing compound, dissociates large P-TEFb complexes [115]. Although these three compounds affect p53 induction in a similar way, they may utilize different mechanisms to influence other biological processes. While the mechanism for the camptothecin-mediated disruption of large P-TEFb complexes remains unknown, it is worthwhile to determine the role of HEXIM1 in p53 activation induced by camptothecin. Importantly, HEXIM1 knockdown cells would not respond to treatment of p53 inducing reagents (*i.e*., flavopiridol and doxorubicin) and did not up-regulate the expression of p53 downstream targets. This unmistakably emphasizes the significance of HEXIM1 in p53 activation following these treatments [113].

Claudio *et al.* found that P-TEFb bound to the *C*-terminal domain (a.a. 361–393) of p53 and phosphorylated p53 at serine 392 [116]. In another report, Radhakrishnan and co-worker adopted the mass spectrometry technique to demonstrate that CDK9 phosphorylated p53 at Ser-33 and Ser-392 [117]. Phosphorylation of Ser33, Ser315, and Ser392 enhances the DNA binding ability and induces transactivation of p53 [118,119,120]. Overexpression of HEXIM1 was found to maintain the phosphorylation of p53 at Ser-33 and Ser-392, and activate the expression of p53 downstream targets, p21 and PUMA [113]. The p53-P-TEFb and p53-HEXIM1 interactions may be two independent events. It is possible that P-TEFb may phosphorylate p53 first. After dissociating with P-TEFb, the phosphorylated p53 binds to HEXIM1, which further stabilizes the phosphorylation of p53 at Ser-33 and Ser-392. Although it has not been determined in our report whether P-TEFb actually participates in p53 phosphorylation, nevertheless, there is a likelihood that other kinases are involving [109,121,122].

## 6. Conclusions

Both p53 and P-TEFb are essential cellular regulators. Generally, p53 is involved in all adult cancers with about 50% of the cancer patients acquiring p53 mutations while the other half is due to the suppression of p53 functions [104]. P-TEFb regulates most transcription by RNAP II and its kinase activity is tightly guarded by HEXIM1. Our studies in the past few years establish the functional connection between these two important pathways. NPM and HDM2 regulate P-TEFb activity through the modulation of HEXIM1, while HEXIM1 induces p53 activation by enhancing its stability. The novel discovery of HEXIM1 in regulating p53 suggests a molecular mechanism for p53 activation induced by anti-cancer drugs/compounds through the interaction with HEXIM1. HEXIM2, a protein sharing extensive homology at the central and *C*-terminal regions of HEXIM1, exhibits similar functions in regulating P-TEFb activity [30,32,33]. It is noteworthy that the central and *C*-terminal domains of HEXIM1 are required for the interaction with p53 [113]. Future investigation is required to examine the involvement of HEXIM2 in the p53 pathway. These discoveries will impart better understanding on the molecular actions of p53-inducing agents which may lead to potential new strategies development for cancer therapy.
